# Online health literacy resources for people with intellectual disability: protocol for a grey literature scoping review

**DOI:** 10.1136/bmjopen-2024-088509

**Published:** 2024-11-24

**Authors:** Zhenmei Yeap, Jessica Keeley, Rachel Skoss, Susan Hunt, Angela Kickett, Jacinta Saldaris, Thomas Nevill, Jenny Downs

**Affiliations:** 1 School of Psychological Science, The University of Western Australia, Perth, Western Australia, Australia; 2 The Kids Research Institute Australia, The University of Western Australia, Perth, Western Australia, Australia; 3 Institute for Health Research, The University of Notre Dame Australia, Fremantle, Western Australia, Australia; 4 Curtin University, Perth, Western Australia, Australia

**Keywords:** Health Literacy, Developmental neurology & neurodisability, Health Equity

## Abstract

**Abstract:**

**Introduction:**

People with intellectual disability are at risk of poor physical and mental health. Risks to health are compounded by poor health literacy, that is, reduced capacity to access health services, respond quickly to changes in health status and navigate care pathways. Building health literacy skills is a strength-based way to increase health and optimise the use of healthcare services. The internet is a primary source of health information for many people, including people with intellectual disability and their families. This scoping review will aim to identify and collate online lay healthcare resources available to and developed for people with intellectual disability and their families and evaluate whether domains of health literacy are addressed.

**Methods and analysis:**

This review will be reported following the Preferred Reporting Items for Systematic Reviews and Meta-Analyses extension for scoping reviews guidelines. The proposed search strategy has three components. Resources will be identified by (1) reviewing disability organisation websites, (2) searching key disability and health terms in the Google search engine and (3) snowball sampling to identify additional resources through links in identified websites and resources. Resources will be selected if they are freely available, presented in or translatable into English, provide health information and are directed to people with intellectual disability or their family members. Extracted data will include descriptors of the source, format, area of health and targeted age range. Content relevant to domains of health literacy will be documented and gaps in available health information will be identified. Study findings will be presented in narrative, tabular and visual forms.

**Ethics and dissemination:**

Ethical approval will not be sought because primary data will not be collected. The results will be disseminated in peer-reviewed literature, as conference presentations, as a synthesised resource for people with intellectual disability and their families and in summary documents for health service managers and policymakers.

**Strengths and limitations of this study**

- The methodology of this study will provide a framework for future grey literature research.
- The methods are informed by Arksey and O’Malley’s guidelines for scoping reviews.
- The rigorous search strategy has three components: (1) targeted organisation website search, (2) Google search and (3) snowball sampling.
- The results will not be a complete sample but will be an extensive representation of health resources available for people with intellectual disability and their families.
- It is possible that people with intellectual disability access health information developed for the general public; however, we only include information that is targeted or designed to be accessible for people with intellectual disability.

## Introduction

 People with intellectual disability experience difficulties with conceptual, social and practical adaptive skills.^1^ Alongside, there are difficulties in developmental and adaptive functioning domains and there is a high prevalence of poor physical and mental health.^2^,^4^ For example, many people with intellectual disability live with sleep disturbances.^5^ Mental health symptoms in children are above the clinical threshold by approximately 38%.^4^ People with intellectual disability have a greater risk of hospitalisation depending on the severity of intellectual disability.^6^ Evidence from Canada and Australia shows that many of these hospitalisations, such as for ear infection, are potentially preventable compared with those without intellectual disability.^7 8^ Health problems^9^ and high rates of potentially preventable hospitalisations persist from as early as childhood.^7 8^ There are urgent needs for healthcare reforms that champion preventive and coordinated healthcare to ensure people with intellectual disability achieve optimal health and quality of life and reduce hospital burden.^10^ For example, the Australian Government has indicated areas of urgent attention to resolve health disparities in the *National Roadmap for Improving the Health of People with Intellectual Disability*.

Health literacy is the ability to access, understand, apply and use health information to meaningfully contribute to one’s health and healthcare.^11^,^14^ When individuals have knowledge of the factors that influence their health and healthcare, their capacity for better understanding and management is maximised. Families with poor health literacy find it difficult to access and engage with services, have poor health knowledge and limited capacity for self-care and caring for the health of others.^13^ Families with socioeconomic disadvantage often have poorer health literacy, and hospital services rather than primary care services can be their medical ‘homes’.^15^ The concept of health literacy also refers to clinical and organisational capacities to support community health.^11^ People with intellectual disability often face additional barriers to accessing health information^16^ as relevant and accessible information can be difficult to find.^17^,^19^ We acknowledge that health literacy supports need to be inclusive and supportive of people with intellectual disability and their families. If service providers have limited skills and/or time to respond to varied health literacy needs across different levels of disadvantages,^13^ inequities for supporting healthcare if needs are complex, such as in intellectual disability, are amplified.

### Rationale

Given that poor health literacy predicts potentially preventable hospitalisations^20^ and that health literacy is a modifiable determinant of health,^21^ building health literacy for individuals with intellectual disability, caregivers and service providers is recognised as a strength-based and affordable approach to improving the health of people with intellectual disability.^13^ Developing and evaluating health literacy interventions is an emerging field, for example, associated with better uptake of cancer screening and treatments.^22^ However, there has been little focus on the needs of vulnerable populations,^23^ including intellectual disability where there have been no clinical trials testing the impacts of health literacy interventions.^24^ There are no peer-reviewed data on what strategies effectively target the needs of people with intellectual disability at different levels of severity, and their families/caregivers.

Many governments, healthcare, disability service providers and advocacy organisations have developed resources to support the health of people with intellectual disability that are available online. However, there is a large amount of information on the internet and end-users vary in their internet searching skills and abilities to identify reliable information that can support them to learn and use relevant health information. Numerous resources have been developed with funding from government sources (eg, Information, Linkages and Capacity Building grants from the Australian Government Department of Social Services) but there is no centralised database of deliverables. Accordingly, knowledge of resources derived from a systematic search to enable the compilation of resources may be helpful.

### Aim

The aims of this scoping review are threefold: (1) to systematically scope and review the online health non-peer-reviewed literature (‘grey literature’), (2) to identify how the information relates to health literacy domains and (3) to identify information gaps that could be addressed.

## Methods and analysis

To map what online health resources are available to people with intellectual disability and their families/caregivers, the methodological framework for scoping reviews by Arksey and O’Malley^25^ will be adapted. This methodology was considered the most appropriate because of its ability to chart the range of information, be flexible and demonstrated utility through its extensive use in the literature. Adaptations to this framework will account for the exclusive inclusion of online health resources as opposed to peer-reviewed or other types of grey literature (eg, thesis, government report). For example, a traditional scoping review would search academic literature databases, whereas in this study Google and specific website searching will be conducted. These adaptations will be informed by two previously published grey literature protocol papers.^26 27^ After identifying the research question, we will search the internet and select relevant studies, extract and chart data, and report results. This review reporting will be guided by the Preferred Reporting Items for Systematic Reviews and Meta-Analyses extension for scoping reviews (PRISMA-ScR).^28^

A grey literature review of online health resources for people with intellectual disability was considered appropriate because of the lack of evidence-based literature on supporting health literacy,^23^ and because multiple resources would be needed across the broad range of health areas. A preliminary search of MEDLINE, the Cochrane Database of Systematic Reviews, Open Science Framework and JBI Evidence Synthesis was conducted prior to the development of the protocol using relevant search terms (health AND resource AND online AND “intellectual disability”) and no current or underway systematic reviews or scoping reviews on the topic were identified.

### Review questions

1. What online health literacy resources are available for people with intellectual disability and their families/caregivers?
2. What health domains are addressed in available resources?
3. What components of health literacy are addressed in available resources?
4. How accessible is the information for people with intellectual disability?

### Inclusion and exclusion criteria

Online resources will be included if they are freely available and provide health literacy information that is related to the health of people with intellectual disability across the lifespan and directed to the person with intellectual disability or their family/caregivers. Resources directed to people with developmental disability or low literacy skills that are not associated with intellectual disability, such as those presented in Easy Read writing style^29^ will be included; however, these will not be searched for specifically. Easy Read or Easy English texts are classified by short simple sentences (often dot points) that present key information, explain difficult terms and are accompanied by images. Plain English text is short sentences and paragraphs that use everyday language and avoid jargon.^29^ These definitions will be used by ZY when determining resource presentation format. Downloadable resources such as guides, fact sheets, book chapters and digital tools such as online modules, applications and webpages will be included in this review. Non-English language documents will not be specifically sought but pages with a translation function will be documented, although we note that language and culture are deeply intertwined and that the Google Translate facility may not fully bridge this gap. Due to limited resources, our review will not include resources exclusively published in languages other than English. Health resources developed for the general public, health practitioners and videos (eg, YouTube) and general social media content will be excluded unless presented in Easy Read or Plain English and included on an identified organisation’s webpage. We will not search for healthcare resources for specific rare diseases with overlapping intellectual disability because we anticipate that parents would search patient advocacy group webpages for condition-specific information. These resources were considered beyond the scope of the aims of the study as although they might be accessed by people with intellectual disability, they are unlikely to be used or helpful due to their complexity. Resources were not excluded based on quality.

### Search strategy

Two searches will be conducted. The targeted search is expected to generate an initial dataset. The second Google search will complement the first by casting a wider and broader net in terms of relevancy and geography. The inclusion of multiple grey literature searches, including a targeted and Google was informed by a protocol published by Brennan and colleagues.^27^

First, a targeted set of Australian government and non-government websites related to intellectual disability, health and advocacy organisations will be compiled by the investigator team drawing on health literacy (RS, JD), caregiver (RS, SH) and disability (JK, RS, JD) expertise. This search will be restricted to Australian organisations to understand what Australian families would find if they searched for health information. Consequently, the results will represent the availability of online health resources. The initial list will include organisations that the research team believe will have health information for people with intellectual disability, including disability organisations, intellectual disability organisations and condition-specific organisations (eg, Inclusion Australia, Down Syndrome Australia). The list will be expanded as new sources of information are identified when searching.

All websites will be individually searched for relevant health resources using their built-in tabs and search functions. For example, some websites might have a ‘health’ or ‘resource’ tab on the homepage, while other websites may have a search function where terms such as ‘health’ and ‘wellbeing’ can be searched. Snowball sampling will involve searching for additional resources that are identified through weblinks and citations within identified resources.

Second, an advanced Google search using key terms will be conducted in incognito mode on Google Chrome. Incognito mode will be used to prevent previous online activity from affecting the search results. This second search will attempt to ensure relevant resources will not be missed and an opportunity to identify international resources. Search terms have been pilot-tested and refined to optimise retrieval of relevant results. This process involved trialling different search terms and identifying those that captured the most relevant results. An initial list included additional condition-specific terms (eg, Down syndrome, Fragile-X syndrome, Prader-Willi syndrome, Rett syndrome, foetal alcohol spectrum disorder); however, the search string did not appear to retrieve a range of relevant resources. The final search string will be “intellectual disability”, health, OR “health literacy”, OR mental, OR physical, OR “wellbeing”. The first 100 results will be reviewed in line with the strategy outlined by Brennan *et al*.^27^ The search terms are presented in table 1. These searches will be completed by the end of October 2024.

**Table 1:** Google advanced search strategies and key phrases used to search for online resources

Keywords and phrases (combined with AND)	
1	“Intellectual disability”
2	health, OR “health literacy”, OR mental, OR physical, OR “wellbeing”
Limited to the first 100 results available in English	

### Resource selection

Websites identified in the targeted searches, advanced Google and through snowball sampling will be screened for relevant resources. Each individual resource will be recorded from identified websites. This will assist us to achieve our goal of producing a resource that is wide-ranging and can be navigated by health topics. A spreadsheet will be created within Excel to record the title, source organisation and URL for the resource. This will be done by one reviewer (ZY). The full text of the included resources will be reviewed by ZY in close consultation with other members of the research team (JK, RS, AK, JD). Reasons for excluding online resources will be documented and reported in the review. The results of the search will be reported in full in the final scoping review and presented in a modified PRISMA-ScR flow diagram.

### Data extraction

All resources will be collated and evaluated in a customised spreadsheet (Microsoft Excel for Microsoft 365 MSO V.2402 Build 16.0.17328.20124), which has been constructed by the research team. The domains and categories have been designed to address the research question and reflect the findings from preliminary searches. Extracted data will include details to describe the organisation, target recipient and content including the application of the health literacy domains (access, understand, apply and use). These categories may change during the process of data extraction if it is additional fields are identified as necessary or if included fields are found to be redundant. The categories of data that will be extracted are presented in table 2. Data charting will be performed by one reviewer (ZY) with extensive consultation with the research team. Uncertainties will be resolved through discussion.

**Table 2:** Categories of data extraction

Domain	Category	Subcategories
Organisation	Name	
	Government or non-government	
	State (if Australian) or country of origin	
	URL	
Resource	Document or page title	
	URL for the specific resource	
	Format	Eg, written, pictorial, video
	Domain of health	Eg, physical health, mental health, general health, female health, sexual health, specific illnesses and diseases, procedures, health system and services, multiple health domains
End-user	Targeted recipient	Eg, people with intellectual disability, family or carers, or people with general disability and age range
Other	References provided	Yes/no
	Translation availability on webpage	Yes/no
	Additional notes	
Health literacy components	Access	Yes/no
	Understanding	Yes/no
	Appraise	Yes/no
	Apply/remember	Yes/no

### Critical appraisal

Each health resource will be rated on whether they address each component of health literacy.^12^ The utility of the information is assessed by applying the domains of health literacy. Working definitions and guiding questions for appraisal created for each component are presented in table 3. To meet the criteria for addressing a component, resources must endorse at least one of the guiding questions yielding a conservative rating. Criteria will be marked and recorded in the spreadsheet by one reviewer. A random selection of ratings will be cross-checked with another investigator and discussed with the team. Additionally, the researcher will have the opportunity to consult with other team members if there is any ambiguity or uncertainty as they appraise the data. Furthermore, data will be collected regarding whether the resource included references to identify if the information presented is evidence-based. No other assessment of quality will be applied as the quality of the source of information is not a focus of this study.

**Table 3:** Working definitions for the components of health literacy and guiding questions for evaluation

Component of health literacy	Working definition^12^	Guiding questions
Access	The ability to seek, find and obtain health information.	How easy is the resource to find? (eg, utility of search function, availability of access via Easy Read hyperlinks or QR codes).
Understand	The ability to comprehend health information.	Are complex words defined? Does it have sufficient language accessibility? Eg, Plain or Easy English format. Has the resource been tested with end-users?
Appraise	The ability to interpret, filter, judge and evaluate health information.	Does the resource cater to different health presentations or groups? Eg, Through sub-headings or hyperlinks to specific concerns. Is there an opportunity for the end-user to reflect on what is relevant or important to them? Eg, reflection questions, acknowledgement that advice may vary across circumstances. Is there evidence to support the evaluation of the resource provided?
Apply/remember	The ability to communicate and use the information to make an informed decision to maintain and improve health.	Does the resource provide tools or practical resources that can be used? Does the resource provide an opportunity to reflect on or practice information? Eg, worksheets, checklists, templates, forms, online course and digital application.

### Data presentation

A narrative summary of the findings will be presented, in addition to tables and a figure, which will detail the categories presented in table 2.

### Patient and public involvement

While there are no study participants, this grey literature review will describe information that is intended to be used by people with intellectual disability and their caregivers. Three members of the study team have contributed expertise from lived experience to the protocol design and will contribute to the execution of the protocol (RS, SH, AK). We will expand our liaison with community members to produce a resource that indexes available resources found in the review.

## Ethics and dissemination

No primary data will be collected and ethical approval will not be sought. The results will be disseminated in peer-reviewed literature, as conference presentations, as a synthesised resource for people with intellectual disability and their families, and in summary documents for health service managers, stakeholders including patient advocacy groups for rare diseases with overlapping intellectual disability, and policymakers.

## References

[1] Schalock RL, Luckasson R, Tassé MJ (2021). Intellectual Disability: Definition, Diagnosis, Classification, and Systems of Supports.

[2] Hughes-McCormack LA, Rydzewska E, Henderson A (2017). Prevalence of mental health conditions and relationship with general health in a whole-country population of people with intellectual disabilities compared with the general population. BJPsych Open.

[3] Hughes-McCormack LA, Rydzewska E, Henderson A (2018). Prevalence and general health status of people with intellectual disabilities in Scotland: a total population study. J Epidemiol Community Health.

[4] Buckley N, Glasson EJ, Chen W (2020). Prevalence estimates of mental health problems in children and adolescents with intellectual disability: A systematic review and meta-analysis. Aust N Z J Psychiatry.

[5] Surtees ADR, Oliver C, Jones CA (2018). Sleep duration and sleep quality in people with and without intellectual disability: A meta-analysis. Sleep Med Rev.

[6] Bebbington A, Glasson E, Bourke J (2013). Hospitalisation rates for children with intellectual disability or autism born in Western Australia 1983-1999: a population-based cohort study. BMJ Open.

[7] Balogh R, Brownell M, Ouellette-Kuntz H (2010). Hospitalisation rates for ambulatory care sensitive conditions for persons with and without an intellectual disability--a population perspective. *J Intellect Disabil Res*.

[8] Weise JC, Srasuebkul P, Trollor JN (2021). Potentially preventable hospitalisations of people with intellectual disability in New South Wales. Med J Aust.

[9] Lin E, Balogh R, Chung H (2021). Looking across health and healthcare outcomes for people with intellectual and developmental disabilities and psychiatric disorders: population-based longitudinal study. Br J Psychiatry.

[10] Emerson E (2021). Inequalities and inequities in the health of people with intellectual disabilities. Oxford Res Encycl Glob Public Health.

[11] Nutbeam D (2009). Defining and measuring health literacy: what can we learn from literacy studies?. Int J Public Health.

[12] Sørensen K, Van den Broucke S, Fullam J (2012). Health literacy and public health: a systematic review and integration of definitions and models. BMC Public Health.

[13] Batterham RW, Hawkins M, Collins PA (2016). Health literacy: applying current concepts to improve health services and reduce health inequalities. Public Health (Fairfax).

[14] Fleary SA, Joseph P, Pappagianopoulos JE (2018). Adolescent health literacy and health behaviors: A systematic review. J Adolesc.

[15] Seguin J, Osmanlliu E, Zhang X (2018). Frequent users of the pediatric emergency department. CJEM.

[16] Doherty AJ, Atherton H, Boland P (2020). Barriers and facilitators to primary health care for people with intellectual disabilities and/or autism: an integrative review. BJGP Open.

[17] Centers for Disease Control and Prevention (U.S.) (2023). How to develop products for adults with intellectual developmental disabilities and extreme low literacy: a product development tool.

[18] Matin BK, Ballan M, Darabi F (2021). Sexual health concerns in women with intellectual disabilities: a systematic review in qualitative studies. BMC Public Health.

[19] Squiers L, Lynch MM, Holt SL (2023). Building Evidence for Principles to Guide the Development of Products for Adults with Intellectual and Developmental Disabilities and Extreme Low Literacy-A Product Development Tool. Healthcare (Basel).

[20] Palumbo R (2017). Examining the impacts of health literacy on healthcare costs. An evidence synthesis. *Health Serv Manage Res*.

[21] Nutbeam D (2009). Building health literacy in Australia. Med J Aust.

[22] Papadakos JK, Hasan SM, Barnsley J (2018). Health literacy and cancer self-management behaviors: A scoping review. Cancer.

[23] Cheng C, Beauchamp A, Elsworth GR (2020). Applying the Electronic Health Literacy Lens: Systematic Review of Electronic Health Interventions Targeted at Socially Disadvantaged Groups. J Med Internet Res.

[24] Lindly O, Crossman M, Eaves M (2020). Health Literacy and Health Outcomes Among Children With Developmental Disabilities: A Systematic Review. Am J Intellect Dev Disabil.

[25] Arksey H, O’Malley L (2005). Scoping studies: towards a methodological framework. Int J Soc Res Methodol.

[26] Braithwaite J, Zurynski Y, Ludlow K (2019). Towards sustainable healthcare system performance in the 21st century in high-income countries: a protocol for a systematic review of the grey literature. BMJ Open.

[27] Brennan L, Brewster L, Lunn J (2023). How do hospitals address health inequalities experienced by children and young people: a grey literature scoping review protocol. BMJ Open.

[28] Tricco AC, Lillie E, Zarin W (2018). PRISMA Extension for Scoping Reviews (PRISMA-ScR): Checklist and Explanation. Ann Intern Med.

[29] Centre for Inclusive Design (2020). A guide to creating accessible content. https://centreforinclusivedesign.org.au/wp-content/uploads/2020/04/Easy-English-vs-Plain-English_accessible.pdf.

