# Supplementary material for: Online health literacy resources for people with intellectual disability: protocol for a grey literature scoping review
Source: BMJ Open. 2024 Nov 24;14(11):e088509. doi: 10.1136/bmjopen-2024-088509 (PMC11590844; doi:10.1136/bmjopen-2024-088509)
Supplement: Supplementary file 1 [file bmjopen-2024-088509.reviewer_comments.pdf]

PEER REVIEW HISTORY

BMJ Open publishes all reviews undertaken for accepted manuscripts. Reviewers are asked to complete a checklist review form and are provided with free text boxes to elaborate on their assessment. These free text comments are reproduced below.

ARTICLE DETAILS

Title (Provisional)

Online health literacy resources for people with intellectual disability: protocol for a grey literature scoping review

Authors

Yeap, Zhenmei; Keeley, Jessica; Skoss, Rachel; Hunt, Susan; Kickett, Angela; Saldaris, Jacinta; Nevill, Thomas; Downs, Jenny

---

VERSION 1 - REVIEW

**Reviewer**	**1**
**Name**	**Bruland, Dirk**
**Affiliation**	**Bielefeld University of Applied Sciences**
**Date**	**28-Jul-2024**
**COI**	**There are no conflicting or competing interests.**

Thank you very much for this high interesting review protocol. The aim of the contribution is to describe a scoping review with a focus on online health information websites and evaluate them accoding to the health literacy model. The topic is high relevant. I noticed some suggestions partly in bullet points character.

Generally

Health literacy and wellbeing are broad terms. Is there an assessment or discussion of how many resources are lost if certain diseases are not specifically addressed but only the general term?

A description, if the search has a precise search or a a sensitive search approach would be benefical.

Abstract

In the introduction of the abstract following sentence can be found: “This grey literature scoping review will aim to identify and collate online lay healthcare resources available to and developed for people with intellectual disability and their families and evaluate how they can support health literacy.” The part evaluate how they can support health literacy is

only mentioned in the conclusions. A more precise coordination with the text is required here.

Introduction

In the introduction, a brief explanation of the understanding of health literacy and the connection with intellectual disability is desirable, as many readers are probably not well acquainted with the target group. The explanation by the table comes late in the text.

Rationale & aim

See note in the abstract: Is it only about the presentation of resources? Or how health literacy can be addressed? While the abstract says "how they can support health literacy", the aim is about discovering gaps.

Methods and Analysis

There is no information on when the review will be carried out.

Due to the mentioned notes in introduction and rationale & aim, the research question needs to be more focussed: Is there a main question (aim) and sub-questions or are all questions equally adjacent?

What is adapted? “the methodological framework for scoping reviews by Arksey and

O’Malley20 will be adapted for this grey literature review.”

Please give more details on the “preliminary search of MEDLINE, the Cochrane Database of Systematic Reviews and JBI Evidence Synthesis was conducted and no current or underway systematic reviews or scoping reviews on the topic were identified” for example when did you do this, what are the search words?

Inclusion and exclusion criteria: What about websites that are not specifically made for people with intellectual disabilities but for people with low literacy in general?

Please provide an argument why Health resources developed for the general public, health practitioners and general social media contentwill beexcluded, but if they presented on the organisation’s webpage they will be included.

Search strategy

How do you know the websites are searched first? And what is about websites in English from the UK, USA (e.g. CDC for the corona virus) or Canada?

For the google search terms, that have been pilot-tested and refined to optimise retrieval of relevant results, please add some more details for understanding, how it was conducted. Google shows also videos mainly from youtube? Are they included or excluded?

Why are the first 100 results will be searched, not the first 500 or 1.000 results?

Resource Selection

Is there a quality assessment of the websites? E.g. in youtube everybody without specific knowledge to people with intellectual disabilietes can do videos?

Data extraction

Is the table fixed or can it be adjusted during the process?

Critical appraisal

It seems, that the data evaluation is a qualitative approach, describing the contents of the websites. Can the implementation fulfill the criteria of objectivity and reliability?

Table 3

The question "Is the resource free?" seems to be redundant because this is an inclusion criteria.

Where do you get the information "Has the resource been tested with endusers?". Often it is not mentioned on the websites themselves.

**Reviewer**	**2**
**Name**	**Breau, Genevieve**
**Affiliation**	**University of Greenwich**
**Date**	**02-Aug-2024**
**COI**	**None**

Thank you for this very interesting and important literature review study protocol. I think it is needed and on an important topic. I have the following suggestions:

1. For the methodology, I would like more detail about how you are using Arksey's and O'Malley's framework, and the rationale for this.

2. I would like a definition of what you are defining as easy read, and how will this be determined?

3. I would like a more systematic approach and description of how you will search for relevant health resources from the grey literature.

VERSION 1 - AUTHOR RESPONSE

Reviewer: 1
Dr. Dirk Bruland, Bielefeld University of Applied Sciences
Comments to the Author:
Thank you very much for this high interesting review protocol. The aim of the contribution is to describe a scoping review with a focus on online health information websites and evaluate them

accoding to the health literacy model. The topic is high relevant. I noticed some suggestions partly in bullet points character.

Thank you for your positive comments and assessment of the manuscript.

Generally
Health literacy and wellbeing are broad terms. Is there an assessment or discussion of how many resources are lost if certain diseases are not specifically addressed but only the general term?
A description, if the search has a precise search or a a sensitive search approach would be benefical.
Thank you for your comment on the specificity and brevity of the research search terms. We have added content to the Strengths and Limitations of the Study section that recognises that the search represents available information and whilst extensive, cannot be fully comprehensive. We acknowledge that there are resources for specific communities associated with rare diseases with overlapping intellectual disability that are not part of our search strategy. Our clinical and research experience indicates that most parents will search for health information on specific pages, e.g., patient advocacy groups for their specific disease, for targeted information, but will search generally also. Accordingly, it will be important to include rare disease organisations in our dissemination of findings. We have included this point in the "Inclusion and exclusion criteria" section on page 6. We have broadened our dissemination strategy on page 8 accordingly.

Abstract
In the introduction of the abstract following sentence can be found: "This grey literature scoping review will aim to identify and collate online lay healthcare resources available to and developed for people with intellectual disability and their families and evaluate how they can support health literacy." The part evaluate how they can support health literacy is only mentioned in the conclusions. A more precise coordination with the text is required here.

Thank you for your comment regarding the need to clarify the aims of the study. We have made changes to the wording to make it clear that we are interested in identifying whether the resources address the different domains of health literacy.

Introduction
In the introduction, a brief explanation of the understanding of health literacy and the connection with intellectual disability is desirable, as many readers are probably not well acquainted with the target group. The explanation by the table comes late in the text.

Thank you for your comment regarding the need to explain the relationship between people with intellectual disability and health literacy. We have included addition content in the second paragraph of the Introduction section to make it clearer and earlier in the text.

Rationale & aim
See note in the abstract: Is it only about the presentation of resources? Or how health literacy can be addressed? While the abstract says "how they can support health literacy", the aim is about discovering gaps.
Thank you for your comment regarding inconsistencies in the stated aims of the research. We

have made changes in the Aim, Abstract, and Introduction sections to improve consistency.

Methods and Analysis
There is no information on when the review will be carried out.

Thank you for your comment regarding when the research will be carried out. We have included content that states that data collection will be completed by the end of October 2024.

Due to the mentioned notes in introduction and rationale & aim, the research question needs to be more focussed: Is there a main question (aim) and sub-questions or are all questions equally adjacent?

Thank you for your comment regarding the hierarchy of the research questions. Additional content has been included in the Aim section to make it clear that the three research aims are of equal importance.

What is adapted? "the methodological framework for scoping reviews by Arksey and O'Malley20 will be adapted for this grey literature review."

Thank you for your comment regarding the need for further detail on the adaptation of the methodological framework. Additional content has been included in the first paragraph of the Methods and Analysis section on page 5 to clarify how the framework was adapted including the inclusion of grey literature rather than peer-reviewed literature, consistent with examples from protocol papers of grey literature searchers which guided these changes.

Please give more details on the "preliminary search of MEDLINE, the Cochrane Database of Systematic Reviews and JBI Evidence Synthesis was conducted and no current or underway systematic reviews or scoping reviews on the topic were identified" for example when did you do this, what are the search words?

Thank you for your comment regarding the need to provide additional information regarding the search for similar reviews. An essential component in preparation for this protocol was to determine that a similar review had not already been conducted and published. Additional content has been included in the second paragraph of the Methods and Analysis section including example search terms.

Inclusion and exclusion criteria: What about websites that are not specifically made for people with intellectual disabilities but for people with low literacy in general?

Thank you for your comment regarding the justification for the inclusion criteria. It is not likely that these types of websites would appear in our search unless they were linked to a disability webpage in which case they would be included if they were otherwise relevant. Additional content has been included to clarify this in the Inclusion and Exclusion Criteria section, alongside a reference to the methods described by the Centre for Inclusive Design.

Please provide an argument why Health resources developed for the general public, health

practitioners and general social media content will be excluded, but if they presented on the organisation's webpage they will be included.

Thank you for your comment regarding the justification of the exclusion of resources that were not developed for people with intellectual disability. Additional content has been included in the Inclusion and exclusion Criteria section to clarify that these resources would be excluded if they were not considered accessible to people with intellectual disability because they would be beyond the scope of the aims of the study. An additional limitation was included to the Strengths and Limitations of this Study section to acknowledge that people with intellectual disability may access information designed for the general public.

Search strategy
How do you know the websites are searched first?

Thank you for your comment regarding the order of searches. Additional information has been included at the beginning of the Search Strategy section to clarify that the searches will be conducted in that order because the first was expected to generate more data and the second was broader and complementary to the first. Our sequential approach was progressively snowballing.

And what is about websites in English from the UK, USA (e.g. CDC for the corona virus) or Canada?

Thank you for your comment regarding the exclusion of international disability organisations. Additional content has been included in the second paragraph of the Search Strategy section to clarify that we are interested in local resources in the first instance before expanding to international searching. We anticipate that results will be representative of online information from multiple countries but will not be a comprehensive world-wide list.

For the google search terms, that have been pilot-tested and refined to optimise retrieval of relevant results, please add some more details for understanding, how it was conducted. Google shows also videos mainly from youtube? Are they included or excluded?

Thank you for your comment regarding the pilot-testing and inclusion of YouTube content. Videos were not identified in the preliminary results. This may indicate that information tends to be presented in text form and not using other modalities such as video. However, we do expect that videos will be embedded in website resources. Additional content has been included in the Inclusion and Exclusion Criteria to clarify this.

Why are the first 100 results will be searched, not the first 500 or 1.000 results?
Thank you for your comment regarding the search results restrictions. We have included additional content that explains with references that this strategy was employed in previous research at the end of the Search Strategy section.

Resource Selection
Is there a quality assessment of the websites? E.g. in youtube everybody without specific knowledge to people with intellectual disabilities can do videos?

Thank you for your comment regarding the evaluation of website quality. Additional content has been included at the beginning and the end of the Critical Appraisal section to clarify that health literacy is a measure of quality in terms of its utility. Further, we have included data on whether the resource presented references as an indicator that the information is evidence-based. However, no additional evaluation of website quality was conducted. Further, quality evaluation was not a focus in this research and is additionally not common practice in scoping reviews.
Data extraction
Is the table fixed or can it be adjusted during the process?
Thank you for your comment regarding the flexibility of the data extraction table. Additional content has been added to the Data Extraction section to explain that the table will be flexible throughout the data extraction process.

Critical appraisal
It seems, that the data evaluation is a qualitative approach, describing the contents of the websites. Can the implementation fulfill the criteria of objectivity and reliability?
Thank you for your comment about the trustworthiness of the analysis process. Additional content has been included to the Critical appraisal section on pages 7 and 8 to further describe our intended trustworthiness processes, e.g., a systematic method for data extraction and consultations between the investigators on the inclusion and description of resources.

Table 3
The question "Is the resource free?" seems to be redundant because this is an inclusion criteria.

Thank you for your comment regarding the criteria for 'access' in Table 1. We agree that this is redundant and so has been removed.

Where do you get the information "Has the resource been tested with end users?". Often it is not mentioned on the websites themselves.
Thank you for your comment regarding information on end-user reviews. We agree that this might not be displayed in the resources. However, this is just one criterion, and it is not necessary to meet them all to demonstrate a domain. It is included as an option to demonstrate part of the domain and to more completely describe Sørensen's integrated model of health literacy.

Sørensen K, Van den Broucke S, Fullam J, et al. Health literacy and public health: a systematic review and integration of definitions and models. BMC Public Health. 2012;12:80.

Reviewer: 2
Dr. Genevieve Breau, University of Greenwich
Comments to the Author:
Thank you for this very interesting and important literature review study protocol. I think it is needed and on an important topic.

Thank you for your positive comments regarding the importance of this study.

I have the following suggestions:
1. For the methodology, I would like more detail about how you are using Arksey's and O'Malley's framework, and the rationale for this.

Thank you for your comment on the need for further information regarding the use of Arksey and O'Malley's framework. Additional information has been included in the first paragraph of the Methods and Analysis section to clarify the rationale and process of using Arksey and O'Malley's framework. Please see our response above for this similar point made by Reviewer 1.

2. I would like a definition of what you are defining as easy read, and how will this be determined?

Thank you for your comment regarding the need to include a definition of Easy Read. We have included a referenced definition of both Easy Read and Plain English and described how they will be applied to the data in the Inclusion and exclusion criteria section. We acknowledge that these types of assessments require some judgement, however, the work is supported by the inclusion of individuals with extensive disability research experience and lived experience as a parent of children with intellectual disability.

Centre for Inclusive Design. A guide to creating accessible content. 2020; https://centreforinclusivedesign.org.au/wp-content/uploads/2020/04/Easy-English-vs-Plain-English_accessible.pdf. Accessed 12/4/2024.

3. I would like a more systematic approach and description of how you will search for relevant health resources from the grey literature.
Thank you for your comment on the need for further detail regarding the grey-literature search. Additional information and clarification has been included throughout the manuscript including the Abstract, Methods and Analysis, Inclusion and Exclusion Criteria, Search strategy, and Resource Selection sections of the manuscript. Your point is similar to the points raised by Reviewer 1 and we hope that our responses are satisfactory.

VERSION 2 - REVIEW

**Reviewer**	**1**
**Name**	**Bruland, Dirk**
**Affiliation**	**Bielefeld University of Applied Sciences**
**Date**	**17-Oct-2024**
**COI**	

Thank you very much for editing the protocol. All comments were addressed. I wish you every success with your literature research.

**Reviewer**	**2**
**Name**	**Breau, Genevieve**

Affiliation University of Greenwich

Date 20-Oct-2024

COI

I have reviewed the comments from the author, and after reviewing the revised manuscript I am confident that my feedback has been addressed.
